# Determining the Depth of Injury in Bioengineered Tissue Models of Cornea and Conjunctiva for the Prediction of All Three Ocular GHS Categories

**DOI:** 10.1371/journal.pone.0114181

**Published:** 2014-12-10

**Authors:** Michaela Zorn-Kruppa, Pia Houdek, Ewa Wladykowski, Maria Engelke, Melinda Bartok, Karsten R. Mewes, Ingrid Moll, Johanna M. Brandner

**Affiliations:** 1 University Medical Center Hamburg-Eppendorf, Department of Dermatology and Venerology, 20246 Hamburg, Germany; 2 Jacobs University Bremen gGmbH, School of Engineering and Sciences, 28759 Bremen, Germany; 3 Henkel AG & Co. KGaA, 40589 Düsseldorf, Germany; University of Florida, United States of America

## Abstract

The depth of injury (DOI) is a mechanistic correlate to the ocular irritation response. Attempts to quantitatively determine the DOI in alternative tests have been limited to *ex* *vivo* animal eyes by fluorescent staining for biomarkers of cell death and viability in histological cross sections. It was the purpose of this study to assess whether DOI could also be measured by means of cell viability detected by the MTT assay using 3-dimensional (3D) reconstructed models of cornea and conjunctiva. The formazan-free area of metabolically inactive cells in the tissue after topical substance application is used as the visible correlate of the DOI. Areas of metabolically active or inactive cells are quantitatively analyzed on cryosection images with ImageJ software analysis tools. By incorporating the total tissue thickness, the relative MTT-DOI (rMTT-DOI) was calculated. Using the rMTT-DOI and human reconstructed cornea equivalents, we developed a prediction model based on suitable viability cut-off values. We tested 25 chemicals that cover the whole range of eye irritation potential based on the globally harmonized system of classification and labelling of chemicals (GHS). Principally, the MTT-DOI test method allows distinguishing between the cytotoxic effects of the different chemicals in accordance with all 3 GHS categories for eye irritation. Although the prediction model is slightly over-predictive with respect to non-irritants, it promises to be highly valuable to discriminate between severe irritants (Cat. 1), and mild to moderate irritants (Cat. 2). We also tested 3D conjunctiva models with the aim to specifically address conjunctiva-damaging substances. Using the MTT-DOI method in this model delivers comparable results as the cornea model, but does not add additional information. However, the MTT-DOI method using reconstructed cornea models already provided good predictability that was superior to the already existing established *in* *vitro/ex* *vivo* methods.

## Introduction

To date, the rabbit Draize eye irritation test [1] is still the only OECD-approved test for the prediction of all three GHS categories for eye irritation in one single test system [2]. In the past, a number of ex vivo and in vitro methods have been developed in order to replace the Draize test. For example, tests either based on isolated animal eyes, like the Bovine Corneal Opacity and Permeation (BCOP) test and the Isolated Chicken Eye (ICE) test [3], [4], [5], [6], or cell-based assays [7], [8], [9], [10], [11], [12] have been described. Furthermore, approaches have been published that take advantage of the reactions evoked by chemicals in incubated hen eggs [7] or invertebrates [13], [14]. Also a test system based on the perturbation and denaturation of corneal proteins which is supposed to mimic the disruptive effect of ocular irritants [15] and various 3D cornea epithelial models [16], [17], [18], [19], [20], [21], [22] have been developed. Currently some methods have gained regulatory acceptance for selected GHS categories. For example, both the BCOP and the ICE test method have been implemented at OECD level to screen for corrosives and severe eye irritants (Cat. 1) on the one hand and for non-classified chemicals on the other hand [23], [24]. In the European Union, the HET-CAM (Hen’s Egg Test Chorioallantoic Membrane) and the Isolated Rabbit Eye (IRE) test have also been accepted for the identification of severe eye irritants [25]. In addition, the Cytosensor Microphysiometer test method has gained validation status for the identification of severe irritants (limited to water-soluble materials) and non-classified substances (limited to water-soluble surfactants and surfactant- containing mixtures) and is now the subject of a draft OECD guideline [26]. All available methods reveal their strengths preferably in the accurate identification of either severe eye-irritants or non-irritants. Test methods which reliably distinguish the mild/moderate irritatants (Cat. 2) from Cat. 1 and the non-irritants (No Cat.) directly, are not yet available. Therefore many of these test methods are intended to be used only within the framework of an integrated testing strategy, either in a top-down or in a bottom-up approach [8], [27], [28].

According to an expert group [27], only methods based on ocular tissues comprising both, the epithelium and the stroma, are thought to allow discrimination between all three GHS categories. This perception is based on studies by Jester and Maurer [29], [30], [31], [32], [33], [34] who showed that the surface area and depth of initial corneal injury (DOI) in epithelium and stroma of rabbit eyes strongly correlate with the eye-irritating potential of chemicals which had been topically applied to the eye. The conclusions of the authors are based on in vivo rabbit studies using live/dead assay in combination with the low volume eye test (LVET) as well as on ex vivo studies on isolated rabbit eyes [33].

In the presented study we aimed to establish an in vitro test method which reliably predicts the eye-irritation potential of chemicals for all three GHS categories within one test. For this purpose the previously established and well characterized reconstructed cornea model, consisting of a collagenous stroma covered with a multilayered corneal epithelium [35], [36], [37], [38], [39], was used. Recently we had demonstrated reproducible production of the cornea equivalent model according to standard operation procedures as well as successful method transfer to other laboratories [40]. Furthermore, the corneal tissues had been treated with 20 chemicals of different eye-irritating potential and physical properties under blind conditions, and the relative viability of the whole tissue was used as endpoint to assess the performance and limitations of the models in two independent laboratories. The best-suited prediction model was based on a 60 minutes exposure period with the test substance and a cut-off value of 40% relative viability to discriminate between non-classified and GHS category 1/2 chemicals. The data revealed a high inter-laboratory concordance in predictivity. However, only those chemicals belonging to GHS Cat. 1 were classified correctly in both laboratories, while 3 out of 15 chemicals belonging to No Cat. or Cat. 2 had been concordantly misclassified in both laboratories.

Therefore it was our aim to increase the predictive capacity of the 3D cornea-based test system in order to eliminate false negative responses on the one hand and to discriminate between all 3 GHS categories in one stand-alone test system on the other hand. This method refinement was performed by integrating an additional physiologically relevant endpoint, namely the initial depth of injury in both epithelium and stroma, into the eye irritation assessment. We developed a new technique to quantify the initial DOI in the reconstructed cornea tissues by combining the MTT viability assay with cryosectioning procedures (MTT-DOI method). The formazan-free area of metabolically inactive cells in the tissue after topical substance application is used as the visible correlate of the DOI. A panel of 25 chemicals which covered all GHS categories in a balanced manner was tested on the cornea models in order to assess performance and predictivity of the MTT-DOI method. The test results were then compared with high-quality in vivo reference data.

Although corneal damage is the most influential driver of eye irritation for all GHS categories, also conjunctival damage was found to be of importance, particularly as driver of irritation for GHS Cat. 2 classification [41], [42]. Thus, some chemicals are categorized as Cat. 2 mainly due to severity and/or persistence of the damage they produce in the conjunctiva in vivo [43]. Furthermore, even if subclassification within Cat. 2 is not mandatory in the EU, US authorities prescribe to distinguish between mild (Category 2B) and moderate irritancy (Category 2A). A great significance of conjunctiva effects has been claimed in the discrimination of Category 2A versus 2B [44]. However, none of the already validated in vitro methods for eye irritation testing sufficiently addresses the conjunctiva involvement. Therefore we developed a 3D conjunctiva model from immortalized human conjunctival cell lines. The conjunctiva model was characterized and subjected to the MTT-DOI measurements with a panel of 12 chemicals with a focus on the mild and moderate irritating Cat. 2 substances. Results from MTT-DOI measurements with 3D cornea and conjunctiva tissue equivalents were compared and critically discussed.

## Materials and Methods

### Materials

Thiazolyl blue tetrazolium bromide (MTT reagent), sodium hydroxide, sodium bicarbonate, HEPES and sucrose were obtained from Sigma-Aldrich (Schnelldorf, Germany). Calcium chloride, PBS without Ca^2+^ and Mg^2+^ (PBS−), isopropanol, Nunc cell culture inserts (0.5 cm^2^ surface area, 0.4 and 3 µm pore size, polycarbonate) were from Thermo Scientific (Roskilde, Denmark). Rat tail collagen solution was purchased from CellSystems (Troisdorf, Germany). Media 199 and Ham’s F12, as well as TrypLE Express were from Invitrogen (Darmstadt, Germany). For construction of cornea equivalents, Keratinocyte Basal Medium (KBM) with a Bullet-kit and the chemically defined Keratinocyte Growth Medium (KGM-CD) were obtained from Lonza (Basel, Switzerland). To build up the conjunctiva equivalents two different media were used: Keratinocyte serum-free medium was from Invitrogen (Darmstadt, Germany), and supplemented with 25 g/mL bovine pituitary extract, 0.2 ng/ml epidermal growth factor (Darmstadt, Germany), and 0.4 mM calcium chloride (Invitrogen, Darmstadt, Germany). DMEM/F12 (Sigma-Aldrich, Schnelldorf, Germany) supplemented with 10% newborn calf serum (Thermo Scientific, Schwerte, Germany) and 10 ng/mL EGF was used for stratification (stratification medium). Penicillin/streptomycin, PBS- and PBS with Ca^2+^, Mg^2+^ (PBS+) were purchased from Biochrom (Berlin, Germany). Bola Teflon O-rings (with 3 inner and 6 outer diameter) were from Bohlender GmbH (Grünsfeld, Germany).

### Test substances

Most of the test materials used in this study were chosen from the database published by the European Center for Ecotoxicology and Toxicology of Chemicals [43], according to the classification of the Globally Harmonized System [45] based on the in-vivo data obtained with the Draize eye irritation test. Dimethyl sulfoxide (DMSO) was chosen from the database from Laboratoire National de la Santé [3], isopropyl acetoacetate was collected from the ZEBET database [46], and glycolic acid from ICCVAM [47]. From the databases, a total of 25 test chemicals were chosen (see Table 1). PBS+ and iso-propanol were used as negative control (NC) and batch control (BC), respectively.

**Table 1:** List of the reference chemicals used in this study.

Chemical	CAS No	In vivo GHScategory	Datasource	Supplier	Physical state	Chemicalclass
Glycerol^a^	56-81-5	No Cat.	ECETOC	Roth	liquid	alcohol
PEG-400^a^	25322-68-3	No Cat.	ECETOC	Sigma	liquid	surfactant
DMSO^a^	67-68-5	No Cat.	LNS	Merck	liquid	surfactant
Toluene	108-88-3	No Cat.	ECETOC	Sigma	liquid	aromatic
3-methoxy-1,2-propanediol	623-39-2	No Cat.	ECETOC	Sigma	liquid	alcohol
2-heptanone	110-43-0	No Cat.	ECETOC	Sigma	liquid	ketone
n-bromohexane	111-25-1	No Cat.	ECETOC	Sigma	liquid	haloginated hydrocarbon
Isopropyl acetoacetate^a^	542-08-5	Cat. 2B	ZEBET	Sigma	liquid	ester
3-chloropropionitrile^a^	542-76-7	Cat. 2B	ECETOC	Sigma	liquid	nitrile
Glycolic acid (10%)	79-14-1	Cat. 2B	ICCVAM	Sigma	solid^b^	acids
2-methyl-1-pentanol^a^	105-30-6	Cat. 2B	ECETOC	Sigma	liquid	alcohol
Ammonium nitrate	6484-52-2	Cat. 2A	ECETOC	Sigma	solid	inorganic
Iso-propanol^a^	67-63-0	Cat. 2A	ECETOC	Roth	liquid	alcohol
Acetone^a^	67-64-1	Cat. 2A	ECETOC	Chemsolute	liquid	ketone
Ethanol^a^	64-17-5	Cat. 2A	ECETOC	Merck	liquid	alcohol
2,6-dichlorobenzoyl chloride	4659-45-4	Cat. 2A	ECETOC	Sigma	liquid	acyl halide
Sodium hydroxyde (1%)	1310-73-2	Cat. 2A	ECETOC	Merck	liquid	inorganic alkalies
Benzalkonium chloride (1%)^a^	8001-54-5	Cat. 1	ECETOC	Sigma	solid^b^	surfactant
Cyclohexanol^a^	108-93-0	Cat. 1	ECETOC	Sigma	liquid	alcohol
Triton X-100 (10%)^a^	9002-93-1	Cat. 1	ECETOC	Sigma	liquid	surfactant
Imidazole	288-32-4	Cat. 1	ECETOC	Sigma	solid	heterocyclic
Quinacrine	69-05-6	Cat. 1	ECETOC	Sigma	solid	heterocyclic
Cetylpyridinium bromide (6%)	140-72-7	Cat. 1	ECETOC	Sigma	solid^b^	surfactant
2-methoxyetyl acrylate	3121-61-7	Cat. 1	ECETOC	Sigma	liquid	acrylate
4-fluoroaniline	371-40-4	Cat. 1	ECETOC	Sigma	liquid	aromatic

The in vivo classification data, chemical and physical properties and suppliers of the reference substances are indicated.

a These chemicals were tested also with 10 min exposure on cornea and conjunctiva models.

b Solid chemicals were tested as liquids. DMSO: dimethyl sulfoxide, PEG-400: polyethylene glycol 400.

### Construction of 3D cornea models

The cornea model consists of a 3D stromal biomatrix with embedded human corneal keratocytes covered with a multilayer of human corneal epithelial cells. The corneal keratocytes (HCK), immortalized with SV-40 adenovirus, were established by Zorn-Kruppa [36] and further characterized by Manzer [38]. The corneal epithelial cells (HCE) were kindly provided by Stephan Reichl from Technical University of Braunschweig, Germany, who received them from the RIKEN cell bank (Tsukuba, Japan). The HCE cells were also immortalized with the SV-40 adenovirus and established by Araki-Sasaki [48]. Both cell lines were cultured as described [40].

The cornea models were prepared according to Engelke [40] with some minor modifications. Briefly, first the stromal layer was prepared in cell culture inserts (3 µm pore size) from collagen embedded HCK cells: six volumes of the collagen solution were gently mixed with one volume of ten-fold concentrated F99 medium (1∶1 mixture of Media 199, Ham’s F12), 2.5 volumes of reconstruction buffer (2.2 g sodium bicarbonate, 4.77 g HEPES and 100 ml 0.5 N sodium hydroxide), and one volume of KGM containing 50,000 HCK cells. Then, 150,000 HCE cells suspended in 300 µl of KGM medium were plated on top of each stroma. The constructs were cultured submerged for 6 days. Then the models were cultivated at air liquid interface for further 7 days to induce multilayer formation.

### Construction, barrier function and immunohistochemical characterization of conjunctiva models

The human conjunctival epithelial cells (ConjEp-1/p53DD/cdk4R/TERT, shortly named HCjE), were kindly provided by Ilene Gipson from Schepens Eye Research Institute at Harvard Medical School in Boston, Massachusetts, USA. The cells were taken from a human bulbar conjunctiva, immortalized by expression of hTERT and characterized by Gipson and coworkers [49]. HCjE cells were cultured as described [49]. The collagenous stroma matrix of the conjunctiva equivalents was produced according to the method described above for the cornea models. Subsequently, 750,000 HCjE cells were placed on top. The equivalents were cultured submerged for two days, and then switched to air-liquid interphase conditions for a culture period of 6 days. Thereafter, stratification medium (see materials) was added topically for 24 h to induce stratification and formation of epithelial tight junctions.

When conjunctiva epithelial models lacking the collagen matrix were used, HCjE cells were directly plated into membrane inserts with 0.4 µm pore size and cultivated as described above for the full thickness models.

Barrier function testing by transepithelial electrical resistance (TER) measurements of conjunctiva epithelial models was carried out using the Endohm chamber and the EVOM resistance meter (World Precision Instruments, Sarasota, Florida).

Immunohistochemistry was conducted as described in [50]. For vertical images, paraffin sections (6 µm) of formaldehyde-fixed tissues were taken. For en-face (horizontal) images, cultures cells grown on membrane inserts were fixed in ice-cold methanol/acetone and processed as described. Primary antibodies against claudin-1 (Cldn-1, 1∶3000), zona occludens protein 1 (ZO-1, 1∶100) and occludin (Ocln, 1∶20), were obtained from Zymed Laboratories (San Francisco, CA) and cytokeratin 13 (CK-13, 1∶80) was purchased from Santa Cruz Biotechnology (Heidelberg, Germany). Alexa Fluor 488 Fab (Life technologies, Darmstadt, Germany) was used as secondary antibody (1∶600). An Axiophot II microscope (Zeiss, Göttingen, Germany) and the software Openlab 2.0.9 (Improvision, Coventry, UK) were used for the evaluation of stainings. All images of stainings from a series of experiments were acquired and processed at the same settings, and representative areas were photographed.

### Test protocol for 3D cornea and conjunctiva models

The cornea models were treated topically with 50 µl of the liquid test substances described in Table 1, for an exposure time of 10 or 60 minutes at room temperature. Since conjunctiva tissues generally showed tendency to contract and shrink during construction, a limiting Teflon ring was fixed on the epithelium of these models with vaseline to avoid leakage of the applied chemicals. Proportionally, the applied volume of test substances were reduced to 10 µl. Solid test substances were applied topically onto the epithelium of the tissues using an oval, 6 mm Volkmann bone curette (Wittex, Simbach Germany), which was calibrated with a defined volume of about 50 mg sodium chloride. For each test substance, as well as for the NC and BC, 3 tissue models per batch were used. To prove good reproducibility of the data, all the test substances were tested in at least 3 separate batches. Following exposure, the tissues were extensively washed with PBS+, and transferred into 1.5 ml MTT solution (1 mg/ml) and incubated for 2 h at 37°C and 5% CO_2_. Then sucrose solution was added to a final concentration of 20% and incubated for another 60 min. Thereafter the tissues were removed from the inserts, transferred into cryomolds and embedded in cryomatrix. The tissues were placed at 4°C for 30 minutes and then frozen in liquid nitrogen. The frozen tissues were stored at −20°C prior to cryosectioning.

### Cryosectioning of the MTT stained tissues and photo-documentation

The cornea and conjunctiva models were sectioned with a Leica CM3050 cryostat at a chamber temperature of −23 to −25°C and object temperature of −19 to −21°C. The thickness of the cryosections was 30 µm. 3 sections per sample from the center of the models were taken for further analysis. For long time preservation, the cryosections were mounted in Fluoromount G. For evaluation, a Leica DMLS binocular transmitted light microscope (Wetzlar, Germany) equipped with a Leica EC3 digital imaging camera and LAS EZ software were used.

### Quantitative analysis of the MTT-DOI

**Figure 1:**
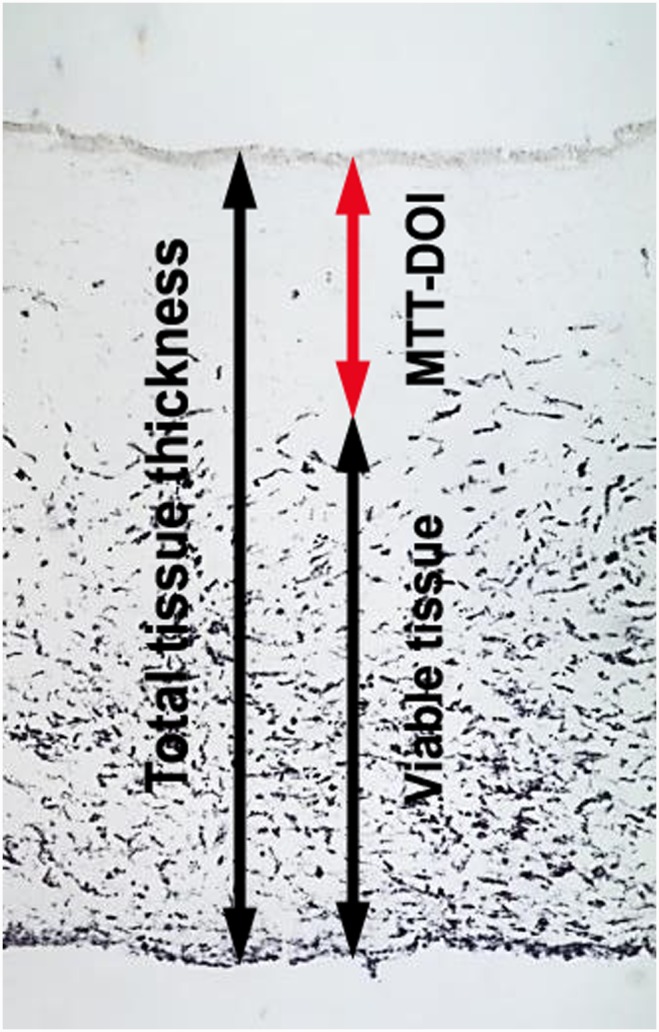
Determination of the DOI from MTT stained cornea tissues after chemical exposure. The MTT-DOI is calculated in relation to total tissue thickness. The presented model was exposed to isopropanol for 60 minutes.

For the quantitative analysis of cell damage and for the determination of the DOI in both tissue models, the images of the sections were processed in ImageJ open source software [51], [52]. After a conversion of the number of pixels into mm, the total cross sectional lengths and the Formazan stained tissue lengths were measured 5 times per image using the straight line tool of the program. The rMTT-DOI was calculated as percentage of the non-viable tissue thickness, where no Formazan staining is present, of the total tissue thickness, as shown in Fig. 1.

## Results

### Development of the DOI-method based on cryosections of MTT-stained 3D-cornea equivalents

We developed a method to analyze the DOI by combining the MTT viability assay with cryosectioning techniques and computer-aided analysis with the aim to establish a prediction model to discriminate all three GHS classes for eye irritation within one model.

When applying common cryosectioning procedures to the bioengineered 3D-cornea models, insufficient tissue preservation was a general issue, in particular induced by shattering due to the high water content of the artificial collagenous stroma. To avoid these freezing artifacts, the unfixed MTT-stained tissues were saturated with 20% sucrose before embedding in cryomatrix [53]. Cutting at approx. −20°C gave the best results for this watery tissue. Adjusting the thickness in the range of 25 to 35 microns was most suitable for sectioning. By using this method it was possible to detect the formazan crystals in the cryosections and to define a clear demarcation between dead and viable tissue areas (Fig. 1).

**Figure 2:**
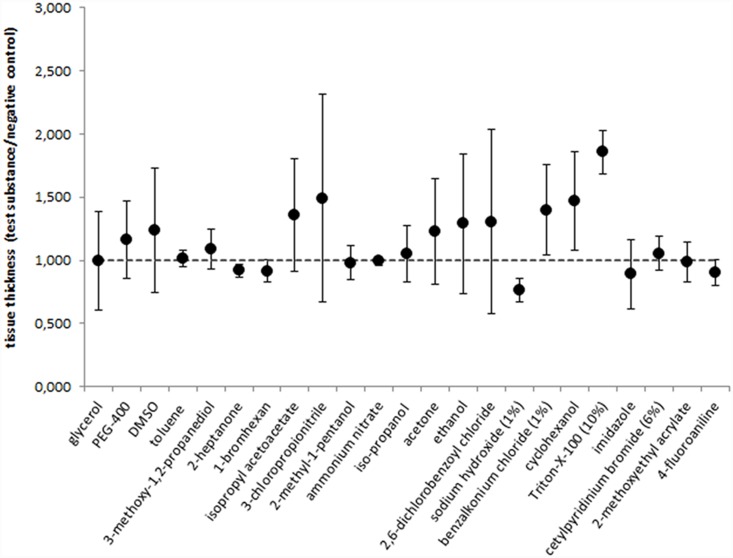
Tissue thicknesses of cornea models after exposure with the chemicals in relation to the thickness of the corresponding negative controls. Means and SDs of 3 batches (3 models per batch).

When testing the reference chemicals we observed that the overall tissue thickness of cornea models was influenced by the test substances itself during 60 min exposure (Fig. 2). This was especially obvious for 1% sodium hydroxide or 10% Triton X-100. Whereas sodium hydroxide led to contraction of the whole tissue, Triton X-100 caused tissue enlargement by swelling. In addition, the total tissue thickness of the NC models was found to vary between the different batches (mean value of the NC = 1.65 mm±0.4). Thus, because of this observed inter-batch variability, it is not suitable to use the absolute DOI value. Instead, we used the relative DOI (rMTT-DOI) given as percent of the total tissue thickness which incorporates the variability in total thickness as a basis for out prediction model.

**Figure 3:**
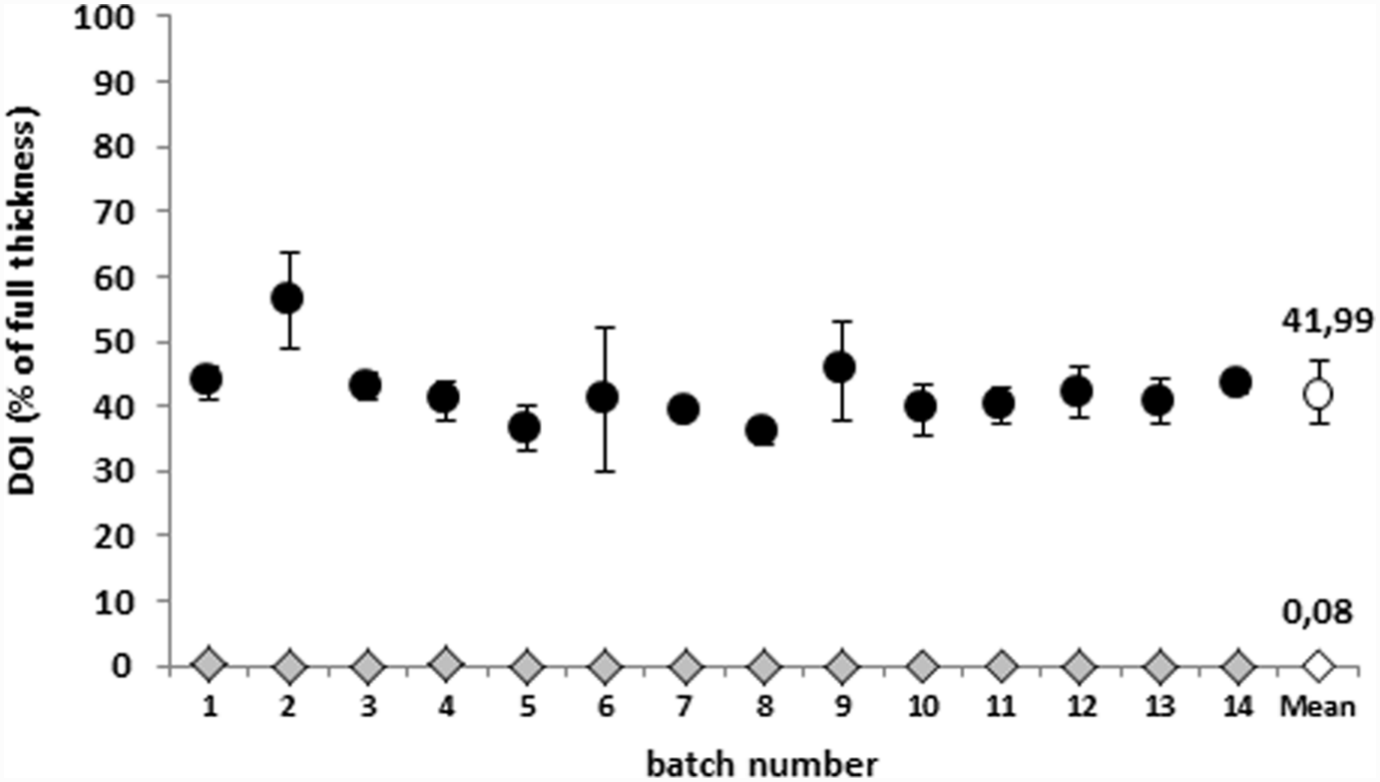
Reproducibility of the rMTT-DOI values for negative controls (rhombs) and batch controls (circles) over 14 runs. Data points represent the means of 3 models. BCs were treated for a 60 min exposure period with iso-propanol, NCs were treated with PBS+ at room temperature.

Fig. 3 summarizes the rMTT-DOI values of NCs and of BCs in the various batches over time. Both values remain relatively constant over time. The NC never exceed values of 0.5% of the total tissue thickness (mean ± SD = 0.08±0.11), whereas rMTT-DOI of the BC vary between 36 and 56% (mean ± SD = 41.99±4.88).

Regarding the epithelial portion of the tissue model, multilayer growth was ensured in all cases.

### Establishment of a preliminary prediction model based on MTT-DOI-measurements of cornea equivalents

**Figure 4:**
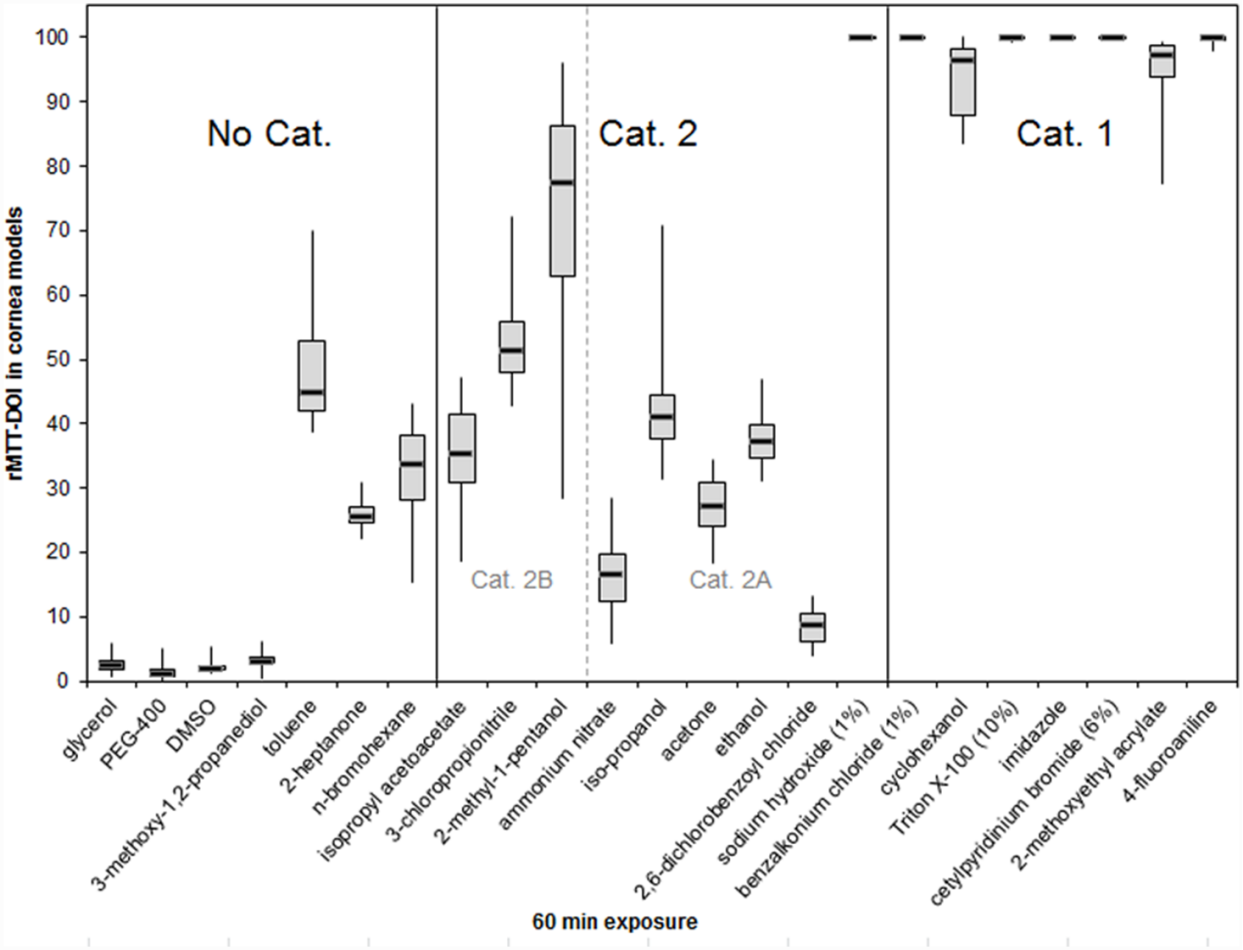
Boxplot analyses showing the distribution of the rMTT-DOI values for 23 of the 25 selected chemicals in corneal models after 60 min exposure. The in vivo GHS categories of the selected chemicals are depicted within the figure. Medians and boxes for upper and lower quartiles are shown. Whiskers indicate minimum and maximum values. (*n* = 9).

**Figure 5:**
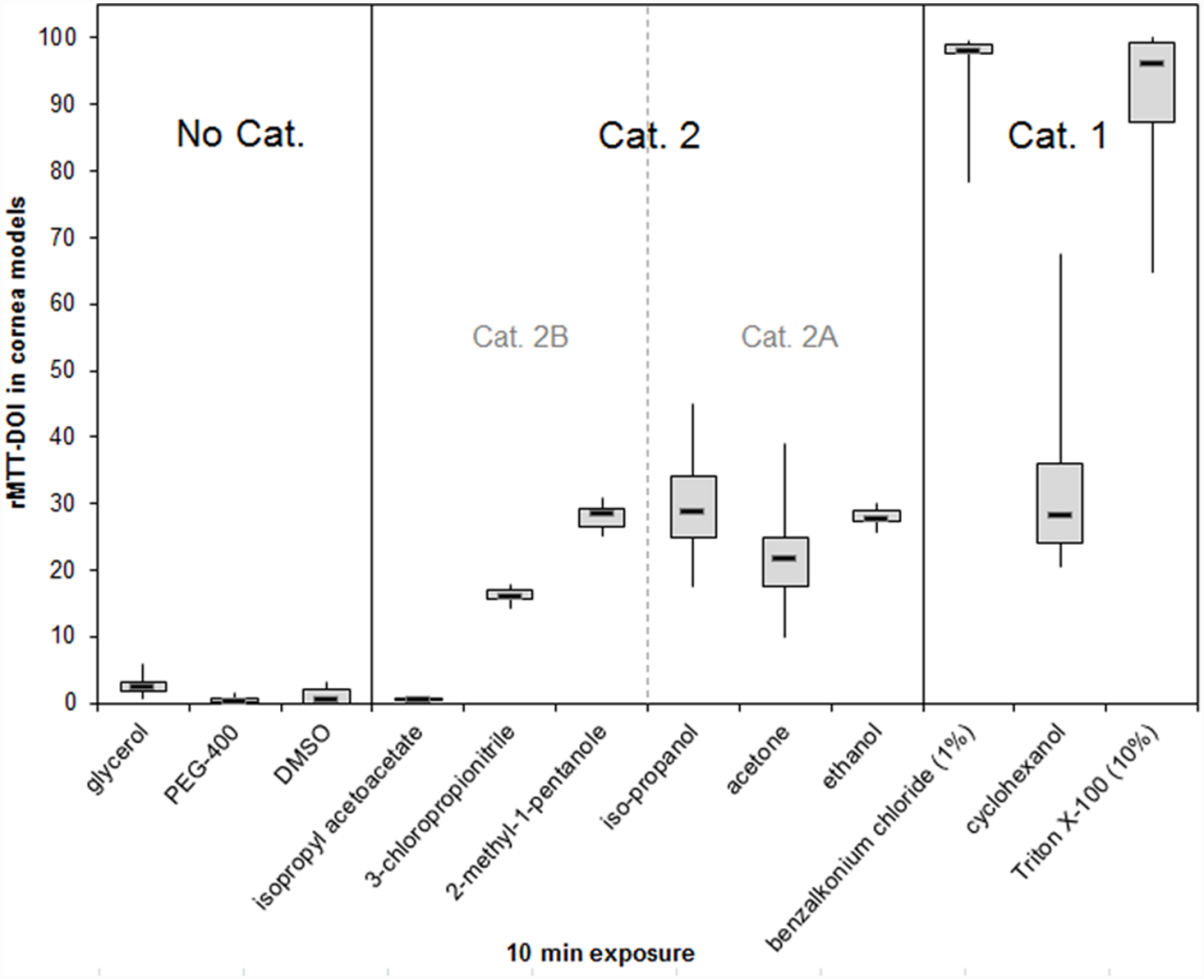
Boxplots presenting rMTT-DOIs in cornea models after 10 min exposure with 12 chemicals with different eye irritating potential. The in vivo GHS categories of the selected chemicals are depicted within the figure. Medians and boxes of 3 batches (3 models per batch) for upper and lower quartiles are shown. Whiskers indicate minimum and maximum values.

To develop an adequate prediction model, 25 chemicals with different physicochemical properties were selected (Table 1). The chemicals cover all categories of eye irritation potential according to the GHS from non-irritant to severe irritant. Adopted from previous studies [40] we used a 60 min exposure times to establish a suitable protocol. In addition, for 12 selected substances also a 10 min exposure period was tested. NCs and BCs were included in each run. Means and standard deviations (SD) of the rMTT-DOIs of three independent batches were calculated (Table 2). In addition, boxplot analyses were prepared (Fig. 4 and 5) to display the characteristic distribution of the rMTT-DOI values for each single test substance in a series of experiments.

**Table 2:** MTT-DOI values in cornea and conjunctiva models after 10 and 60 min exposure with selected chemicals.

Chemical	In vitro MTT-DOI (% of total tissue thickness)						
	Cornea model60 min			Cornea model10 min		Conjunctiva model10 min	
	Mean	SD	In vitro Cat.	Mean	SD	Mean	SD
Glycerol	2.94	0.95	No Cat.	1.64	1.00	0.61	0.3
PEG-400	1.48	0.99	No Cat.	0.46	0.37	0.18	0.22
DMSO	2.31	0.66	No Cat.	1.14	1.18	0.72	0.56
Toluene	48.60	1.78	Cat. 2	-	-	-	-
3-methoxy-1,2-propanediol	3.33	1.08	No Cat.	-	-	-	-
2-heptanone	26.00	0.99	Cat. 2	-	-	-	-
n-bromohexane	32.88	6.59	Cat. 2	-	-	-	-
Isopropyl acetoacetate	35.19	6.86	Cat. 2	0.61	0.21	0.5	0.68
3-chloropropionitrile	53.28	1.23	Cat. 2	16.19	1.17	8.79	4.87
Glycolic acid (10%)	nd	nd		-	-	-	-
2-methyl-1-pentanol	72.07	9.98	Cat. 2	28.10	1.20	18.05	3.76
Ammonium nitrate	16.45	2.33	Cat. 2	-	-	-	-
iso-propanol^a^	41.99	5.29	Cat. 2	29.41	7.09	24.02	8.86
Acetone	26.89	4.59	Cat. 2	21.56	6.70	19.61	5.14
Ethanol	37.48	1.83	Cat. 2	28.11	1.40	15.76	4.95
2,6-dichlorobenzoyl chloride	9.84	1.63	Cat. 2	-	-	-	-
Sodium hydroxyde (1%)	99.25	1.28	Cat. 1	-	-	-	-
Benzalkonium chloride (1%)	99.97	0.03	Cat. 1	96.03	6.46	88.17	18.43
Cyclohexanol	94.03	3.42	Cat. 1	33.81	13.55	28.25	7.21
Triton X-100 (10%)	99.93	0.07	Cat. 1	91.16	12.09	95.39	12.04
Imidazole	99.97	0.03	Cat. 1	-	-	-	-
Quinacrine	nd	nd		-	-	-	-
Cetylpyridinium bromide (6%)	99.98	0.04	Cat. 1	-	-	-	-
2-methoxyetyl acrylate	95.35	3.09	Cat. 1	-	-	-	-
4-fluoroaniline	99.69	0.54	Cat. 1	-	-	-	-

**Figure 6:**
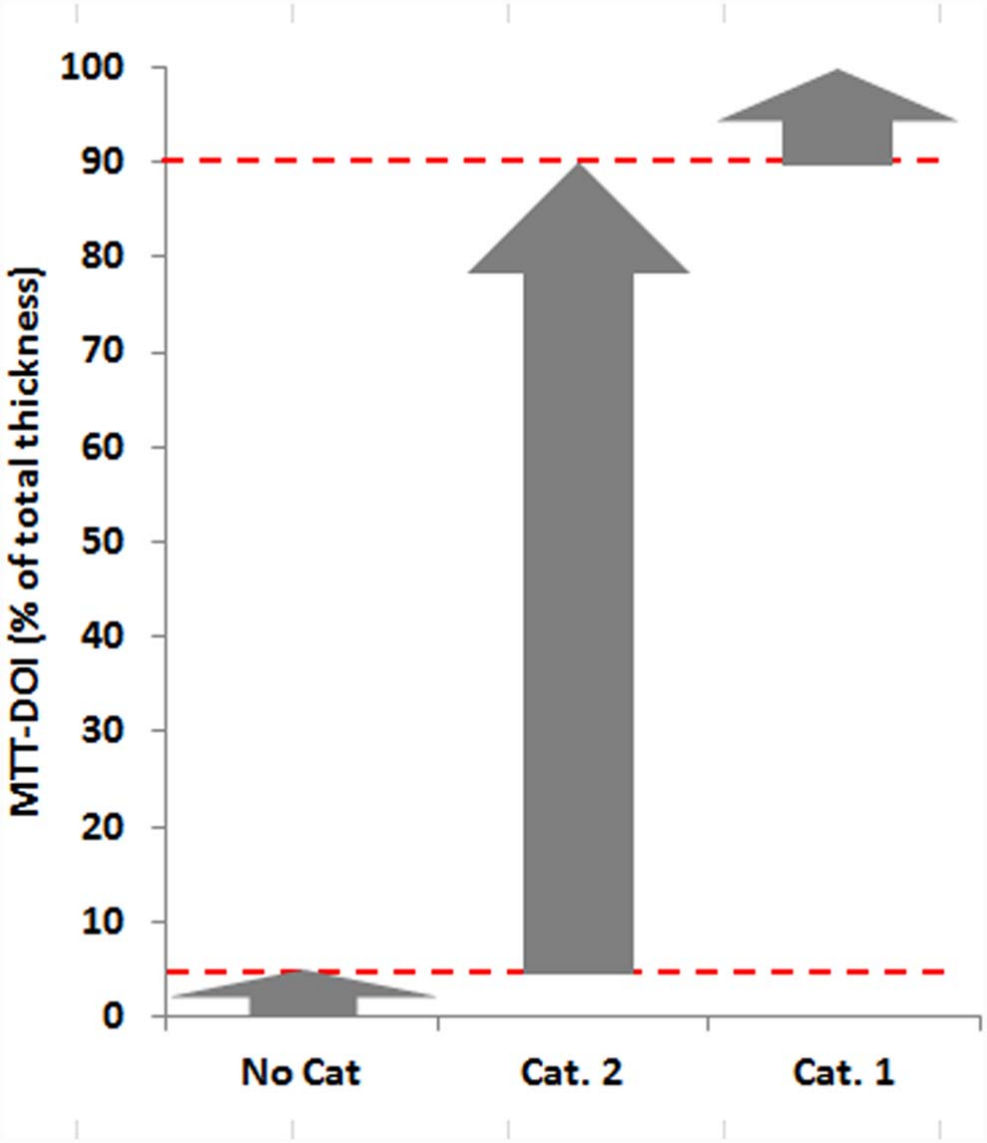
Preliminary prediction model for the MTT-DOI in vitro classification of chemicals, based on the cornea equivalent system and a 60 min exposure protocol.

In vitro scores and classifications were calculated according to the prediction model using a 60 min exposure time, as depicted in Fig. 6. rMTT-DOI means and SDs of 3 individual runs are given.

a Iso-propanol was used as BC and therefore tested in each run. DMSO: dimethyl sulfoxide, PEG-400: polyethylene glycol 400.

Using the 60 min exposition protocol, all rMTT-DOIs found for the Cat. 1 chemicals were higher than 90%, indicating that these chemicals severely damage the cells of the whole tissue models (Fig. 4). A second cluster of compounds is formed by the No Cat. chemicals with rMTT-DOIs below 5% (Fig. 4), indicating that the tissue damage induced by these substances occurs only at the superficial layers and is more or less restricted to the corneal epithelium.

However, when using the 10 min exposure protocol (Fig. 5), we observed that the DOIs of chemicals from different GHS classes cannot be separated satisfactorily. For example, the isopropyl acetoacetate belongs to GHS Cat. 2 but in our 10 min measurements it displays low rMTT-DOI values similar to that of all tested No Cat. substances. In addition, the in vivo severely irritating cyclohexanol possessed a relatively low rMTT-DOI comparable with that of the tested mild and moderately irritating substances. Thus reduction of the exposure time from 60 to 10 min weakens the ability to discriminate between all three GHS categories and would lead to a higher incidence of false negative predictions when incorporated in a prediction model.

When comparing the rMTT-DOI values obtained after both exposure times, most of the substances led to higher values after 60 min than after 10 min. This was most noticeable for the Cat 2B substances.

According to the results of the 60 min exposures a preliminary prediction model was defined using two cut-off values for the in vitro classification of the test chemicals into the three GHS categories as shown in Fig. 6. In this prediction model test chemicals with rMTT-DOIs of ≤5% are assigned to the non-irritant class (No Cat.) and chemicals with rMTT-DOIs ≥90% are assigned to the severe irritant class (Cat. 1). All other chemicals with intermediate rMTT-DOIs are predicted as Cat 2 chemicals.

The outcome of the evaluation of all test substances and their predicted categorization according to the above defined prediction model is summarized in Table 2.

From the 25 substances tested, 19 were categorized correctly. Of the other six substances, toluene, 2-heptanone and n-bromohexane were classified false positive as Cat. 2, and 1% sodium hydroxide false positive as Cat. 1.

For two chemicals no reliable MTT-DOIs could be determined: Glycolic acid led to a complete decomposition of the collagen matrix. Quinacrine resulted in a patchy MTT-formazan stain even in the epithelium, indicating some remaining viability, although Quinacrine seemed to affect the whole tissue.

### Reliability and predictive capacity of the test system

To assess the relevance of the test system, reliability and predictive capacity were determined from a contingency table (Table 3).

**Table 3:** Contingency table showing the in vivo Draize test data versus the in vitro predicted categories, based on the prediction model utilizing the cornea equivalent system and a 60 min exposure protocol.

Category	In vivo Cat. 1	In vivo Cat. 2	In vivo No Cat.	Sum
**In vitro Cat. 1**	7	1	0	8
**In vitro Cat. 2**	0	8	3	11
**In vitro No Cat.**	0	0	4	4
**Sum**	7	9	7	23

Accordingly, using the 60 min exposure times, all Cat. 1 chemicals and 89% of the Cat. 2 chemicals were predicted correctly. Thus the accuracy of prediction for the severe and the mild and moderate irritant substances is very good. Furthermore, no compound was false negative predicted in the MTT-DOI test, but 43% of the non-irritants were overestimated.

In consequence, the overall concordance rate of 83% implies a substantial agreement between the in vitro and in vivo estimates. However, the test system is over-predictive with regard to non-irritant chemicals.

Regarding the physicochemical properties, all alcohols and surfactants and 2 of the 3 solid compounds were correctly predicted. However the pH extreme compounds were over-predicted or led to no result, especially strongly acidic compounds destroy the collagen matrix, as it was found for 10% glycolic acid.

### Successful establishment of 3D conjunctiva equivalent models

With the aim to test its ability to improve predictivity of the 3D cornea model, we established human 3D conjunctiva equivalent models (conjunctiva epithelial model and full conjunctiva model) constructed from human immortalized conjunctiva cells (see materials and methods for establishment).

**Figure 7:**
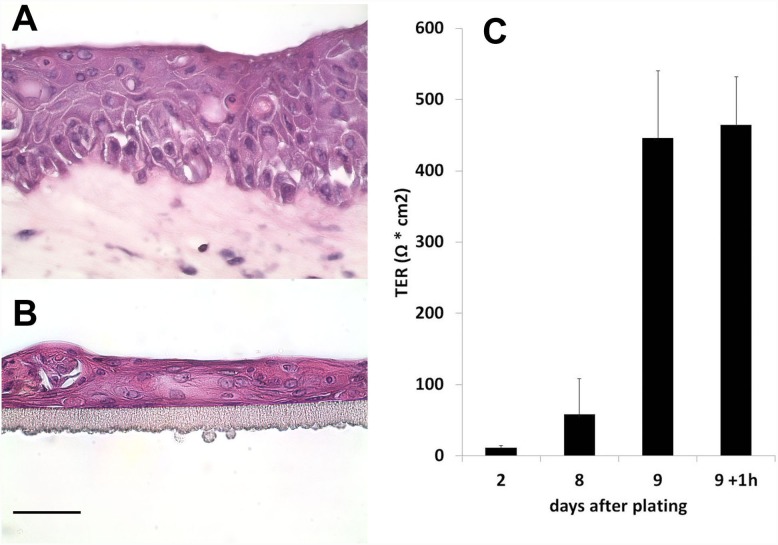
H&E stain of paraffin sections of conjunctiva models at 40-fold magnification (A, B). A shows a conjunctiva full thickness model with stratified epithelial layers on top of the stromal matrix with embedded fibroblasts. B shows a conjunctiva epithelial model without stromal matrix. (Scale bar represents 50 µm.) C: Diagram summarizing the TER values of conjunctiva epithelial models after 2 to 9 days after construction. Note: Measurements on day 8 were just before addition of serum/EGF-containing stratification medium. On day 9 epithelial constructs were measured before and directly after 1 h incubation with PBS+.

Barrier function measurements confirmed an increase of transepithelial resistance in conjunctiva epithelial models after 9 days of cultivation (Fig. 7C). When mimicking chemical exposure conditions by 1 h incubation with PBS+, no significant influence on TER was observed. Thus, the conjunctiva cells are able to form a barrier under these culture conditions.

**Figure 8:**
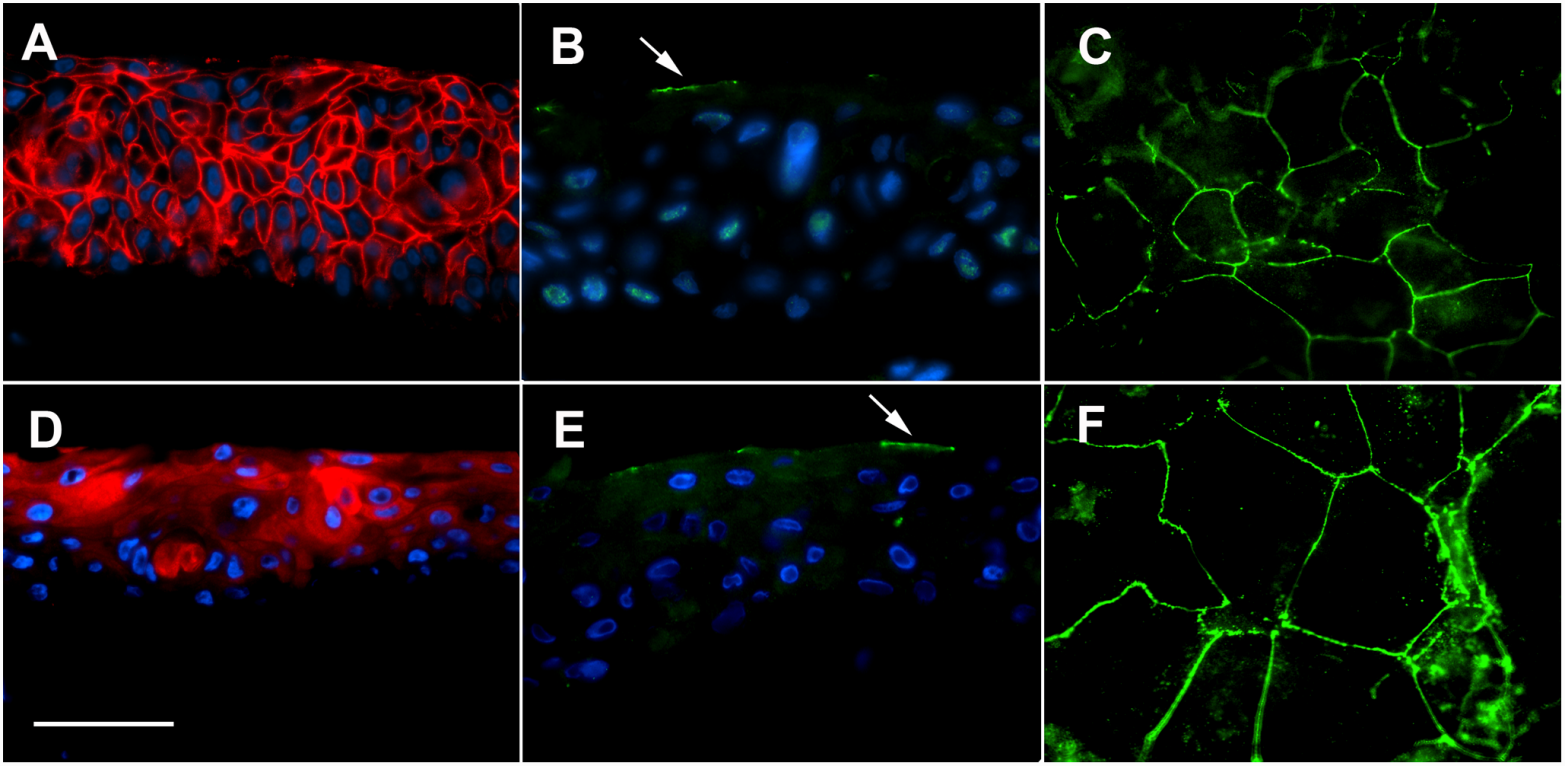
Immunofluorescence localization of tight junction components Cldn-1 (A red), ZO-1, (B, C, green), and Ocln) (E, F, green), and of conjunctiva-specific CK-13 (D, green) in conjunctiva full thickness tissue models. Note: localization of ZO-1 and Ocln is restricted to the apical membrane of superficial epithelial cells (red arrows). A, B, D, E: vertical sections; C, F: horizontal sections. Scale bar represents 50 µm.

The stromal layers of the full conjunctiva models underwent contraction and shrinkage during construction which was even increased during final stratification phase. Therefore measurements of the TER with conjunctiva models did not reveal reliable results due to voltage leakage at the margins of the reconstructed tissues. However, as depicted in Fig. 7A, the 3D conjunctiva models consist of 4–8 layers of epithelial cells, and compared with the epithelial models (Fig. 7B), multilayer formation and stratification was even enhanced on top of collagen matrices. For further biochemical characterization of barrier function in both conjunctiva models expression and localization of the tight junction components Cldn-1, ZO-1, Ocln, and of conjunctiva-specific CK-13 were analyzed. Immunofluorescence images show conjunctiva–characteristic expression of the different proteins in the cross sections of both models (Fig. 8). The microscopic evaluation reveals an overall expression of Cldn-1 and a more apical expression of CK-13 whereas ZO-1 and Ocln are localized at the apical superficial membranes of the epithelial cells.

### MTT-DOI-measurements of conjunctiva equivalents

For reasons of comparison with the corneal model, the MTT-DOI-measurements were only performed with the full conjunctiva equivalents also consisting of epithelium and collagen embedded cells.

For substance application to conjunctiva models we used a limiting Teflon ring affixed with Vaseline in order to avoid leakage of the chemicals since conjunctiva tissues showed tendency to contract and shrink during construction (see also 3.4). When testing these models we generally took a 10 min exposure to minimize the time of usage of the Teflon ring which might affect the measurements.

**Figure 9:**
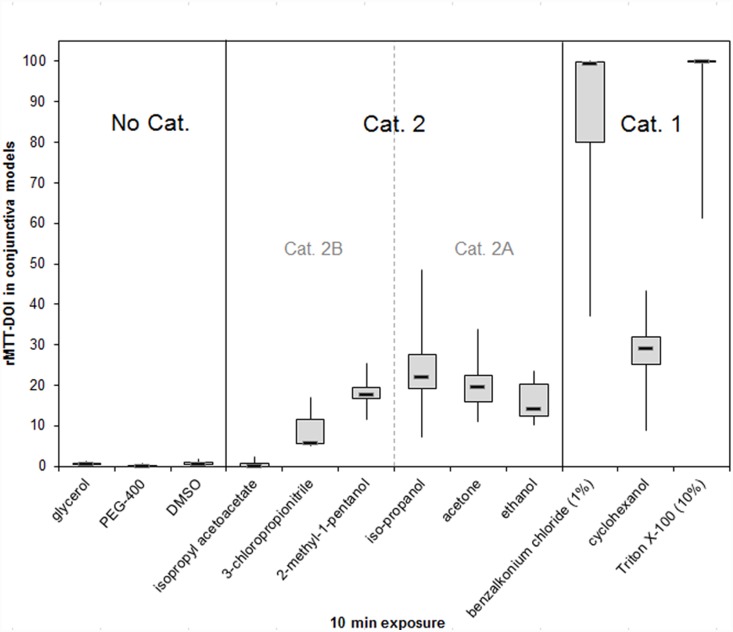
Boxplot analyses presenting the MTT-DOIs generated with conjunctiva models after 10 min exposure with 12 chemicals. The in vivo GHS categories of the selected chemicals are depicted within the figure. Medians and boxes for upper and lower quartiles are shown. Whiskers indicate minimum and maximum values. (*n* = 9).

Twelve reference substances with different physicochemical properties were tested on conjunctiva models. Boxplot analysis of the resulting rMTT-DOI values are depicted in Fig. 9. Means ± SDs of rMTT-DOI values are summarized in Table 2. Quite similar to the studies using cornea models, rMTT-DOIs from Cat 2A and 2B obtained with conjunctiva models lay close together and could not be separated satisfactory. When comparing the rMTT-DOI values obtained with conjunctiva and cornea models after 10 min, it is obvious that both tissues generate very similar results with respect to their susceptibilities towards the selected chemicals of all categories. In particular chemicals which have been shown to react in vivo primarily on the conjunctiva like isopropyl acetoacetate and iso-propanol did not result in higher rMTT-DOI values in conjunctiva models compared to cornea models.

## Discussion

In order to provide a straightforward and reliable method to predict the eye irritation potential of chemicals, we developed a cryosectioning procedure for MTT-stained unfixed 3D cornea equivalents to measure the initial depth of injury in relation to the total tissue thickness. By using this method supplemented by ImageJ-based quantitative analysis tools, we can clearly distinguish reference substances of different GHS categories by means of their rMTT-DOIs.

In contrast to the ex vivo DOI measurements on isolated rabbit eyes (IRE-DOI) described by Jester [33], where classification is based on differential DOI values from both epithelium and stroma, our classification system is exclusively based on DOI in the entire tissue. This modification is due to the fact that in the cornea equivalent stromal cell death almost always occurs only after total loss of epithelial viability. With respect to the choice of a suitable endpoint, MTT reduction to formazan by living cells was preferred over of f-actin/TUNEL staining used by Jester, because the latter, apoptosis-specific staining method failed to give any reliable and reproducible results when adapting it to our cornea equivalent system. This might be due to the different embedding procedures necessary for the native animal corneas on one hand and the artificial stromal tissue on the other hand. However, measurement of MTT-derived formazan offers a valid, simple and widely used method of assessing cell viability. Moreover, contrary to f-actin/TUNEL, which is based on the detection of apoptosis-associated DNA strand breaks, MTT reduction is a marker reflecting viable cell metabolism. Therefore it is independent from the mechanism or timing of the detected cell death which plays a role in TUNEL staining.

In the presented study, 23 of the selected 25 test chemicals caused MTT-DOIs which were detectable with our MTT-DOI/cornea model test system. 10% glycolic acid did not result in any measurable MTT-DOI, because it dissolved the collagenous stromal matrix leading to total destruction of the reconstructed tissue. Since also 1% sodium hydroxide was over-predicted as a severe irritant compound, we conclude that pH extreme compounds (pH<2.0 or >11.5) in general might be overestimated or rather incompatible with the MTT-DOI test in reconstructed cornea models as it has already been described for other in vitro methods [54]. An overestimation of acids and alkalines in our system might be caused by the fact that any pH buffering mucous- and/or tearfilm-producing component, which is present in vivo, is missing. However, it has often been described that alkalines induce chemical burns to the ocular surface resulting in disastrous and challenging injuries in vivo [55], [56], [57].

The second substance which could not be evaluated with our method was quinacrine. In contrast to all the other test chemicals, quinacrine induces cell death in the stroma while parts of the epithelium are still viable. This observation indicates that the impact on the epithelium is not preceding the impact on the stromal keratocytes. Our observation corresponds to the European Commission/British Home Office (EC/HO) validation study which showed that also the BCOP test cannot predict the actual degree or depth of injury for quinacrine [58], [59]. Presumably, this chemical leads to a delayed onset of full degree of irritation.

The investigation of our test substances showed, that all severe irritant substances were categorized correctly. Further this was true for 89% of the mild and moderate irritant substances and 57% of non-irritants. Hence, the MTT-DOI test method promises to be suitable to discriminate between severe irritants (Cat. 1), and mild to moderately irritant Cat. 2 substances, which cannot be covered by any other test system published so far.

According to the ECETOC database, toluene, 2-heptanone and n-bromohexane, belong to the non-irritant GHS category and are therefore over-predicted as mild and moderate irritants using the MTT-DOI method. However, toluene has also been misclassified in other studies: For example, the European Union Risk Assessment Report [60] reported from different eye irritation studies in rabbits where toluene was found to be irritant in 2 of 3 studies [61], [62],. In addition, two studies reported eye irritation in toluene-exposed humans [63], [64],. Also von Burg and coworkers confirmed the eye irritating potential of toluene in rabbits and humans [65]. Eye irritation induced by toluene was also detected with alternative test methods such as the BCOP test [66], [67], the HCE assay [17], and the SMI test [13]. Therefore, the rabbit Draize test data reported in the ECETOC data base [43], are thought to underestimate the irritation potential of this chemical.

Also for 2-heptanone and n-bromohexane contradicting results to the Draize test exist in literature. According to NIOSH Pocket Guide to Chemical Hazards, 2-heptanone is indeed irritating to the eye and for n-bromohexane it was found by Kojima that this substance could only be correctly predicted with modification of the rinsing protocol within their in vitro test system [68]. In addition, n-bromohexane is classified as a skin-irritating substance [69].

Taken into account these fundamental limitations in reliability of predictions due to inconsistent in vivo data, the MTT-DOI test method promises to be suitable to discriminate not only between severe irritants (Cat. 1), and mild to moderately irritant Cat. 2 substances but also identify the non-irritating substances with high accuracy.

It is worth mentioning that no compound was false negative predicted in the MTT-DOI test and no surfactant and no alcohol resulted in a false prediction. Thus, compared to BCOP and ICE tests, the MTT-DOI test promises to cause less false results of alcohols, surfactants as well as ketones [70]. Since two of the three solid chemicals were correctly predicted, the MTT-DOI method is also not necessarily restricted to liquids as it is for example the case for the Fluorescein Leakage test or the Cytosensor Microphysiometer test method [2], [26].

Further, the predictive capacity of the MTT-DOI method for volatile chemicals seems to be higher than in other in vitro systems [67] since all tested volatile chemicals (vapor pressure>6 kilopascal at 25°C), as there are ethanol, acetone and iso-propanol, were correctly predicted.

Compared to the prediction models from our previous studies where spectrophotometric determination of MTT reduction of the whole tissue was used as endpoint, the prediction model based on rMTT-DOI values thus represents a significant and promising improvement.

In our hands, 60 min of incubation showed a more reliable result to categorize the test substances compared to 10 min of incubation. However, in view of quickness of the method, it might be worthwhile testing more substances for this purpose. In addition, the increase of rMTT-DOI values from 10 min to 60 min exposure was most noticeable for the Cat 2B substances, indicating that the exposure time might be a crucial factor for separation of Cat. 2A and 2B.

Although damage to the cornea is the crucial driver of eye irritation for all GHS categories, conjunctiva damage gains more importance particularly as driver of irritation for GHS Cat. 2 classification [41]. Several chemicals are categorized as Cat. 2 mainly due to severity and/or persistence of the damage they produce to the conjunctiva in vivo. However, none of the already validated in vitro methods for eye irritation testing sufficiently addresses the conjunctival involvement. Therefore, it was our aim to develop a bioengineered conjunctiva model in order to explore whether it is possible to close the above-mentioned gap with an additional ocular tissue.

For this purpose the human hTERT-immortalized human conjunctiva epithelial cell line HCjE [49] was used to build up a full thickness and an epithelial conjunctiva equivalent, respectively. An immunohistochemical analysis of both models confirmed the expression of characteristic marker proteins. Cldn-1 staining was observed in all cell layers of the conjunctival epithelium which is in line with Yoshida [71], who found overall epithelial Cldn-1 staining in tissue preparations of human conjunctivas. Regarding ZO-1 and Ocln we found also the same localization at the apical superficial tight junctions described by Yoshida in the native conjunctiva. Also functionality of the tight junctions was confirmed by measurement of TER in the epithelial conjunctiva equivalent. Evaluation of TER in the full thickness conjunctiva equivalent is was not possible due to technical reasons. Furthermore, our immunohistochemical analysis reveal within the epithelium of both models a mostly apical expression of the conjunctiva-specific cytokeratin CK-13 which is in line with Ramirez-Miranda [72] and Paladino [73] who described a similar distribution pattern of CK-13 in the native human conjunctiva and in a bovine 3D conjunctiva model, respectively.

In order to compare the effects of chemicals with conjunctival damage as the main driver of irritation [41] in both the corneal and in the conjunctival model, isopropyl acetoacetate and iso-propanol were selected from ECETOC list among 10 other test chemicals. However, when comparing the effects of these substances on conjunctiva and corneal 3D models, no biologically relevant differences were found in sensitivities towards the chemicals. Unlike the in vivo Draize test, where separation of Cat. 2A and 2B mainly depends on the degree of conjunctiva damage, this was not found in vitro when using 3D conjunctiva models.

A few comparative studies have been published so far in terms of cytotoxicity testing in corneal and conjunctival cultures, dealing mainly with pharmacological relevant compounds like benzalkonium chloride or thiomersal [74], [75], [76]. Due to best availability, most of these studies were undertaken with the Chang’s human conjunctiva cell line [77] and in particular with the Wong-Kilbourne-Derivat of conjunctiva, clone 1-5c-4. Unfortunately, this cell line is described to be HeLa contaminated [78] and therefore it is not recommended to use it any more. Still, in line with our findings, cytotoxicity data generated with these cells did not hint to any biologically relevant differences in susceptibility between corneal and conjunctival cells.

Nonetheless, the conjunctiva full thickness model might be used for other studies, e.g. for basic biological questions using knock-out strategies of specific proteins. In addition, the epithelial conjunctiva model, which was used here mainly as an intermediate step for the generation of full thickness conjunctiva models may be useful, e.g. for the investigation of the influence of substances on barrier function.

## Conclusions

In order to present an adequate, stand-alone strategy for the assessment of all 3 GHS categories of eye irritation we developed a MTT-based method to determine the initial depth of injury in 3D tissue models of the human eye. The test was applied to the previously developed cornea equivalent system and 25 reference chemicals of different GHS categories were tested. Our results reveal that the MTT-DOI test allows us to distinguish between the cytotoxic effects of all GHS categories in one test system. The overall concordance rate of 83% implies a substantial agreement between the in vitro and in vivo estimates. The system is slightly over-predictive with respect to the non-irritant chemicals. The method is currently being tested in a ring trial with a small set of carefully selected chemicals. In order to meet the requirements for additional test systems assessing more specifically the conjunctiva involvement within an eye irritating response, we established a human cell based in vitro full thickness conjunctiva equivalent. The conjunctiva model reveals quite similar to in vivo conjunctiva regarding the initial depth of injury. However, it could not provide additional information to the cornea-based test method even when testing those chemicals which display conjunctiva damage prior to corneal effects, and thus seems to be dispensable for in vitro safety assessment testing. However, it may be beneficial for barrier function analysis and further biological questions.
